# Wearable Devices for Classification of Inadequate Posture at Work Using Neural Networks

**DOI:** 10.3390/s17092003

**Published:** 2017-09-01

**Authors:** Eya Barkallah, Johan Freulard, Martin J. -D. Otis, Suzy Ngomo, Johannes C. Ayena, Christian Desrosiers

**Affiliations:** 1 Laboratory of Automation and 3D Multimodal Intelligent Interaction (LAIMI), Department of Applied Sciences, University of Quebec at Chicoutimi (UQAC), 555 Boulevard de l’Université, Chicoutimi, QC G7H 2B1, Canada; eya.barkallah1@uqac.ca (E.B.); johan.freulard1@uqac.ca (J.F.); martin_otis@uqac.ca (M.J.-D.O.); 2 Laboratory of Automation and 3D Multimodal Intelligent Interaction (LAIMI), Department of Health Sciences, University of Quebec at Chicoutimi (UQAC), 555 Boulevard de l’Université, Chicoutimi, QC G7H 2B1, Canada; suzy_ngomo@uqac.ca; 3 Department of Software and IT Engineering, École de Technologie Supérieure (ÉTS), 1100 Rue Notre-Dame Ouest, Montreal, QC H3C 1K3, Canada; christian.desrosiers@etsmtl.ca

**Keywords:** posture, center of pressure, instrumented insole, IMU, supervised classification, feature selection, neural networks

## Abstract

Inadequate postures adopted by an operator at work are among the most important risk factors in Work-related Musculoskeletal Disorders (WMSDs). Although several studies have focused on inadequate posture, there is limited information on its identification in a work context. The aim of this study is to automatically differentiate between adequate and inadequate postures using two wearable devices (helmet and instrumented insole) with an inertial measurement unit (IMU) and force sensors. From the force sensors located inside the insole, the center of pressure (COP) is computed since it is considered an important parameter in the analysis of posture. In a first step, a set of 60 features is computed with a direct approach, and later reduced to eight via a hybrid feature selection. A neural network is then employed to classify the current posture of a worker, yielding a recognition rate of 90%. In a second step, an innovative graphic approach is proposed to extract three additional features for the classification. This approach represents the main contribution of this study. Combining both approaches improves the recognition rate to 95%. Our results suggest that neural network could be applied successfully for the classification of adequate and inadequate posture.

## 1. Introduction

Several studies, summarized by da Costa et al. [1], have reported that Work-related Musculoskeletal Disorders (WMSDs) generally result from repetitive movements and prolonged or inadequate postures. Pain is the main WMSD symptom, although they may also be accompanied with abnormal motor patterns such as movement deficits (elevation, rotation, etc.) [2] and lack of strength [3], both of which can lead to work disability. Worldwide, the magnitude and prevalence of WMSDs represent a public health concern encountered in most industrialized societies [4,5,6,7,8]. Thus, over 40 million workers in Europe are affected by MSDs attributable to their work [9]. In the province of Ontario (Canada), based on population data of 45,650 individuals aged 16 years and over, MSDs were mentioned as a reason for 40% of all chronic conditions, 54% of all long-term disabilities, 24% of restricted activity days, and almost 20% of health care utilization [4]. The impact of MSDs was even greater in the 65 and over age group [5,10]. According to the Committee on Standards, Equity of Occupational Health and Safety of Quebec (CSEOHS), the number of WMSD lesions affecting workers between 2012 and 2015 represented approximately 27% of the cases claimed, disregarding the non-reported cases [11]. Furthermore, MSDs are responsible for morbidity in many working population and are known as an important occupational problem with increasing health costs. In the last past decade, MSD-related costs were estimated at around 17 billion pounds in the UK, 38 billion euros in Germany, 215 billion dollars in the US, and 26 billion Canadian dollars [12]. Therefore, there is a critical need to introduce effective detection solutions of inadequate postures which may lead to WMSD in the industry.

WMSD detection often focuses on monitoring ergonomic risk factors by self-report questionnaires and/or by direct or indirect observational methods [13,14]. However, it is unclear why on a similar workstation, a person will develop a MSD while another will not. Thus, identifying the personal determinants of MSD development remains a challenge, and a better detection of work-related risk factors is still an important issue [13]. The use of electronic assessment tools seems to be the most promising solution for successful detection [15]. Various technologies, such as 3D motion analysis, have been exploited to gain a more objective or quantitative indication of worker posture and movements [16,17,18]. However, camera-based systems require considerable workspace or time-consuming calibration operations, and their costs are usually quite high. The most commonly used device to analyze human posture is the force platform, which measures displacements of the center of pressure (COP) [19]. COP is defined as the point location of the vertical ground reaction, and is often used to identify a balance deficit [20]. Although these measures are reliable and valid, force platform systems are expensive and reduce the ability to detect in real time the quantitative parameters of postures in real work environment [21,22]. Furthermore, the subject is limited in movements (a quasi-static position). Another approach consists in using sensors attached to the worker for collecting data related to this worker’s own exposure variables [23,24,25]. A possible shortcoming of this approach is its encumbrance, weight, and lack of portability, which limit its use in work activities. For body motions studies, there is a trend towards combining observational approaches with direct recording methods that use instrumentation [18,26]. While combining several detection procedures in a multidisciplinary approach should provide better results than using a single procedure, it is unclear how to combine them for optimal results. Also, implementing these combinations can take a lot of time.

Thereby, in this study, we focus on the design of an effective measurement system for differentiating automatically adequate and inadequate postures. As an electronic and automatic assessment tool, we first introduce an enactive insole and smart helmet as wearable devices to better analyze workers’ posture. Since posture analysis can be both an expensive and time-consuming process, previous studies have underlined the need for automatic models that can detect inadequate posture [27]. Some researchers have demonstrated the relevance of advanced statistical methods such as logistic regression [28], Bayesian models [29], artificial neural networks (ANN) [30] or K-nearest neighbor (KNN) algorithms [31] to predict the risk associated with occupational exposures. These methods have produced varying prediction accuracy, depending on the task factors examined and the quality of the data used for model development. In many cases, neural networks have been shown to yield superior predictive accuracy compared to, for example, multiple linear regression models [32,33]. This could be attributed to the ability of ANNs to model complex and nonlinear relationships between variables [34]. It may also be due to the fact that the statistical methods investigated have rigid assumptions associated with the nature of the data (e.g., linearity, normality, homogeneity of variance), whereas, neural networks make no such assumptions [35]. Thus, we hypothesized that an ANN model could determine effectively the posture of a person in a real working environment, with easily measurable clinical variables such as the COP.

The main contribution of the present study is to develop a new methodology for classifying the current posture of workers using the developed instrumentation. More specifically, we establish a new type of features, related to the COP and inertial measurement unit (IMU) data, to classify postures at workstations. To achieve this goal, we focus on prolonged or inadequate postures, since those can constitute a personal determinant of WMSD-related risk. The proposed classification algorithm is optimized by using a hybrid feature selection method.

## 2. Design of the Measurement System

**Figure 1:**
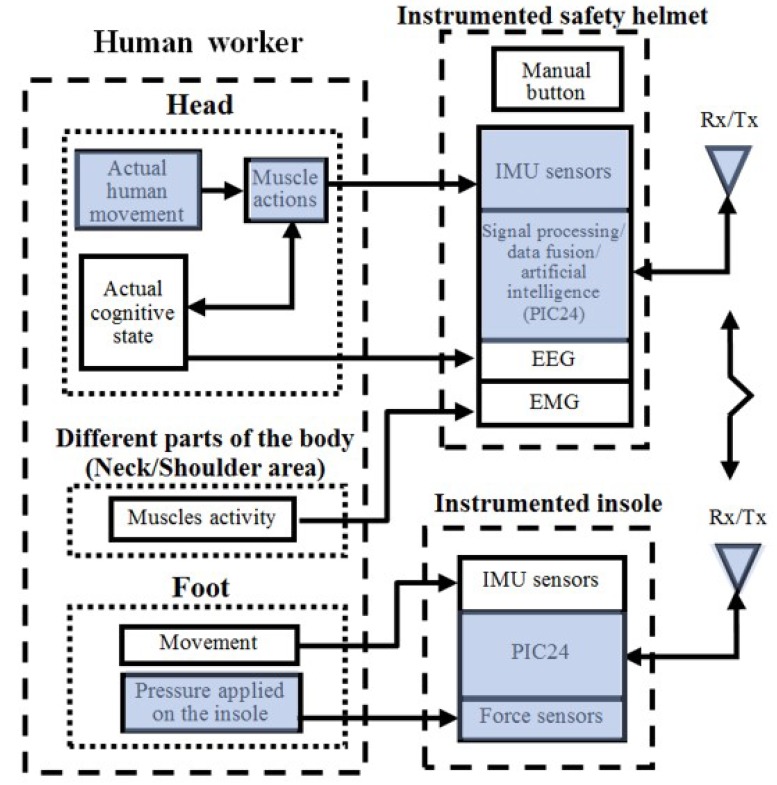
Block diagram of the overall interactive measurement tools (only the grey blocs are analyzed in this study).

The proposed system consists of two assessment tools as wearable devices: (1) an instrumented safety helmet for recognizing the gestures of the worker’s head and (2) an instrumented insole for assessing local COP displacements under the foot. The architecture of these interactive tools is represented in Figure 1. The data recorded by the two assessment tools are sent by wireless transmission (Rx/Tx) to an Android operated device. To effectively analyze worker postures, force sensors are also integrated into an insole for evaluating the pressure applied on the insole and compute the COP. The IMU signals from the helmet is processed in real time by the PIC24 using an embedded Kalman filter.

### 2.1. The Instrumented Safety Helmet

**Figure 2:**
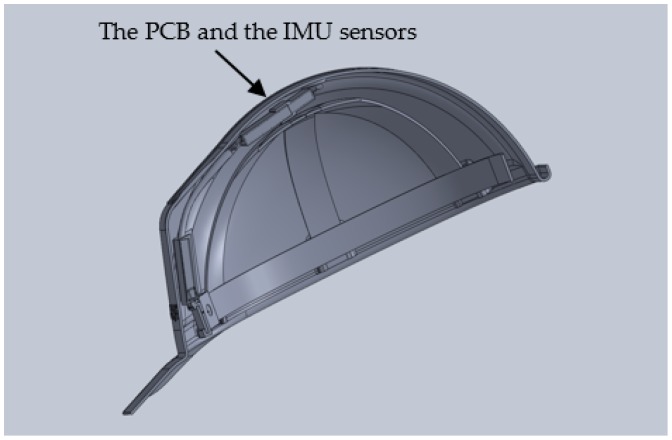
The instrumented safety helmet prototype.

The safety helmet system is an inexpensive, non-intrusive, non-invasive, and non-vision-based system (Figure 2). It includes an IMU that measures the head’s acceleration, velocity, and orientation through a set of 3DOF accelerometers, 3DOF gyroscopes and 3DOF magnetometers. It should be noted that these portable, inexpensive, non-intrusive, non-invasive sensors are increasingly being used in biomechanics and clinical experimentation applications.

The accelerometer adopted and used in the experimental trials of this work is the MPU9250 from TDK InvenSense (San Jose, CA, USA).

### 2.2. Enactive Insole Design

**Figure 3:**
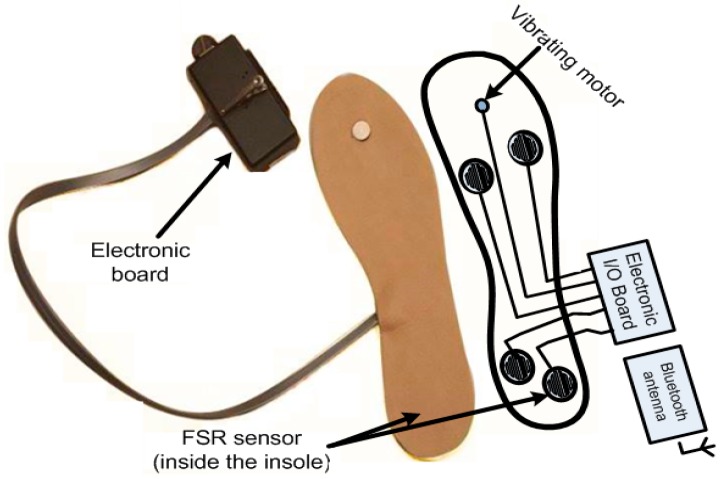
The prototype of the enactive insole with the preferred sensor location (the vibrating motor having the function of a rhythmic pattern is not used in this study).

Worker posture can be analyzed by the COP’s displacement under the foot. This displacement is a virtual site of the plantar surface (Figure 3). More precisely, it is the average location of all the pressures acting on the foot at any given time [36]. Numerous studies [37,38] have examined COP displacements to assess and understand postural control during quiet stance or gait [39]. We have chosen to evaluate the posture and movement of the operator using the COPs positions (barycenter) computed as follows [40,41]:

(1) $$X_{\mathrm{COP}}=\frac{\sum_{i=1}^{n}X_{i}P_{i}}{\sum_{i=1}^{n}P_{i}}\;\mathrm{and}\;Y_{\mathrm{COP}}=\frac{\sum_{i=1}^{n}Y_{i}P_{i}}{\sum_{i=1}^{n}P_{i}}$$

where n denotes the total number of sensors, i denotes a certain sensor, P_i_ is the pressure measured on sensor i, and X_i_, Y_i_ are the COP’s coordinates for sensor i inside the insole.

From these measurements, the challenge is to classify the actual worker’s posture (and movement) into one of several predefined groups of postures (and movements), known as being either adequate or inadequate. To find the COP, we suggest one insole containing four force sensors (Figure 3). Several studies cited in [42,43] have shown that COP does not differ between dominant and non-dominant limbs. It was symmetric in young and healthy adults. Thus, we think that a single insole placed on the right foot (the foot we have chosen as usually the dominant foot of participant) could measure the COP positions in order to acquire the features we need for posture classification. In other studies related to the computation of the risk of fall, we also use only one enactive insole (located on the dominant limb) with successful results [44]. All this makes it possible to reduce the production cost of the device and use a minimal configuration and architecture (already presented in two other studies [45,46]). This allows the classification of the postures, not to optimize this configuration. This system is mainly designed for a long-term monitoring of worker and not for a diagnostic aid tool. Moreover, this system is not adapted for a clinical experimentation or clinical research.

Three sensors could also be used in our proposed insole, however the whole surface under the foot would not be covered. To determine the coordinates of the COP in the anteroposterior (Y_COP_) and mediolateral (X_COP_) directions, we can use the data provided by the four force sensors located in our instrumented insole according to Equation (1). We begin the next subsection by comparing four types of force sensor technologies. Then, a characterization of several types of conductive supports is performed.

#### 2.2.1. Comparison of Force Sensor Technologies

It is known that the force sensing resistor (FSR) sensor allows measuring the center of pressure parameters [41] using Equation (1). Based on previous research works in this field, the number of sensors to compare is high. Therefore, this study only compared three sensors to a FSR sensor that can be added inside a very thin insole. This allowed us to optimize the current consumption in our proposed insole device.

- The capacitive force sensor (CS8-100N, SingleTact, Los Angeles, CA, USA) has the same shape as the FSR sensor. It is ultra-thin, thus allowing its integration in an instrumented insole without any issues. Its consumption is estimated at 2.5 mA according to its specifications [47], which is rather high for a long term usage. Another disadvantage is that it costs three times more than the FSR sensor. Since the target price is around hundred dollars, lower-cost sensors (less or equal to ten dollars each) were investigated.
- Various studies have also investigated the use of an optical sensor [48,49,50,51]. To exploit this sensor for an instrumented insole, we need to integrate a light-emitting diode (LED), a phototransistor and a flexible structure that can introduce an obstruction between the LED and the phototransistor. The load over the structure adjusts the obstruction. The main issue of this technology is the current consumption of the light source, which still needs some improvements.
- The last type of sensor investigated is the Hall Effect sensor. Its operating is based on the shifting of a permanent magnet (N52 3 mm × 1 mm) according to a fixed Hall Effect transistor (177725z, SparkFun Electronics, Boulder, CO, USA). When the magnet is closer to the transistor, the output tension is higher.

**Figure 4:**
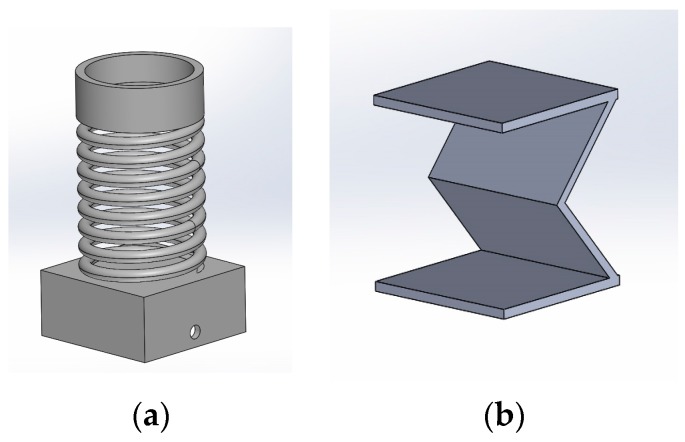
Two different designs for the permanent magnet supports: (**a**) First design; (**b**) Second design.

For the Hall Effect sensor, we first designed two magnet supports (as shown in Figure 4) to incorporate this sensor into the insole during the rubber’s solidification. Figure 4a shows the first version of the magnet support. After the design of this part, it had several defects. Using a spring is more expensive and is somewhat useless in an insole since the Young modulus of the material used in the fabrication of the insole is used instead of the spring Young modulus: the spring Young modulus will not change the measurement. Therefore, the insole material becomes an important choice for this sensor as it would affect the measurement. The complexity of the integration of the permanent magnet near the insole is then complex. Thus, we had to think of a simpler use and design concept. Therefore, the second version of the support of the magnet (Figure 4b) has been conceptualized.

**Figure 5:**
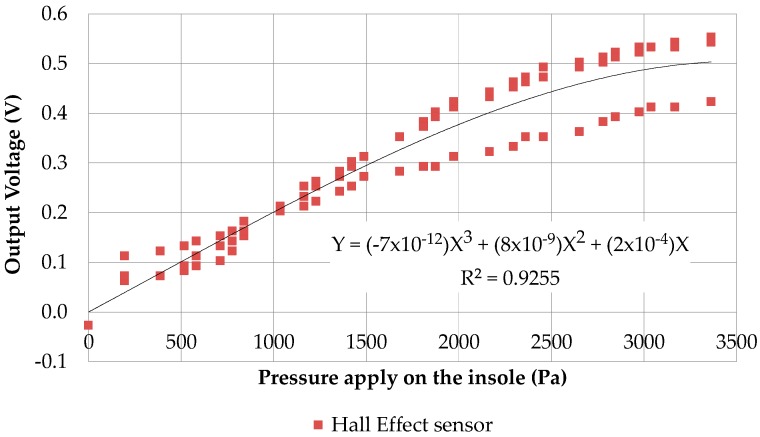
Output voltage of Hall effect sensor with a one millimeter magnet.

The final choice of this design used is only based on practical considerations for the integration of the permanent magnet near the transistor. This simple support only suspends the permanent magnet without considering the Young modulus of this support in order to get very low-cost sensor. Moreover, this support enables to fix the magnet at the top of the insole. The transistor is located at the bottom of the insole and the pressure of the feet will bring closer the magnet and the Hall Effect transistor. With a preliminary calibration, we were able to have an operational sensor. However, before the insole design, different tests were done with an elastomer insole four millimeters thick. The Hall Effect transistor was realized with a 1 mm diameter magnet. The output voltages of this transistor (Figure 5) are no better than those of a 3 mm diameter magnet due to its smaller measuring range.

**Figure 6:**
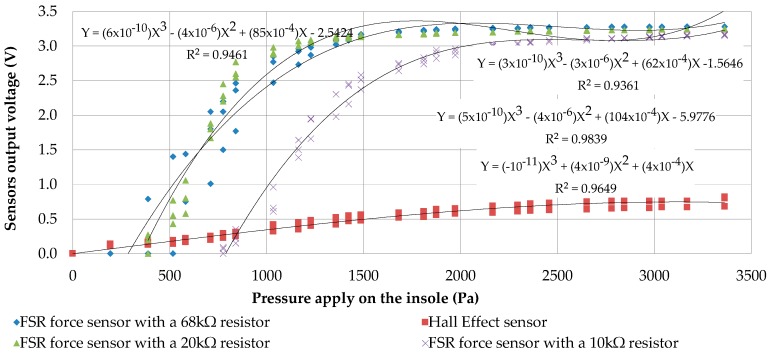
Graphic of force sensors measures with different FSR’s resistor compared to a Hall Effect sensor.

Secondly, in Figure 6, we compared our integrated Hall Effect sensor to the FSR sensor built with different resistors. The current consumption could be reduced on the FSR sensor with a higher resistor for the voltage divider, which gets saturated earlier.

**Figure 7:**
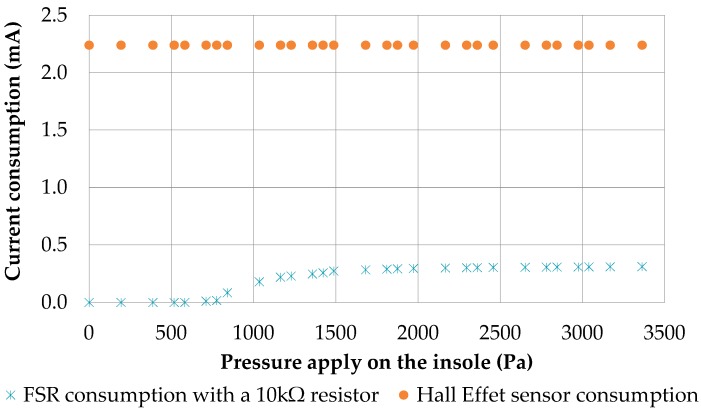
The current consumption of FSR (with 10 kΩ) and Hall Effect sensors.

We note the saturation around 2000 Pa with the Hall Effect sensor and the FSR sensor at 10 kΩ. However, the saturation limit is reached earlier as soon as we increase the resistor for reducing the current consumption. Our measurements suggest the use of a 10 kΩ resistor even if its current consumption is higher (up to 3.3 V). At this resistor value (10 kΩ), the FSR sensor consumes maximally 0.33 mA while the Hall Effect sensor consumes 2.3 mA (Figure 7). Due to its higher consumption, the Hall Effect sensor could not replace the FSR sensor in our system. For this study, we did not find a LED with consumption under 0.33 mA producing enough light to be used. However, a cluster of micro LED or carbon nanotubes are next-generation light sources that could eventually be used in our application. Thus, the FSR seems to be the best to use for our measurement system design (Figure 3).

#### 2.2.2. Characterization of Different Types of Conductive Supports

Classical copper wire could break inside an insole during its use. Conductive textiles provide an interesting solution to prevent this problem. For our insole design, we characterized different types of conductive textiles between an embedded acquisition system (PCB) and the sensors.

**Figure 8:**
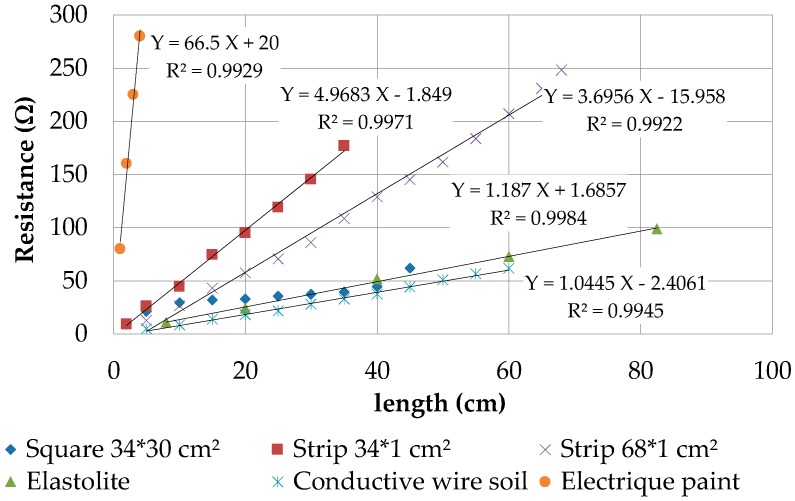
Resistance of conductive supports as function of its length.

To compare the conductivity and find the material best suited for our insole, we characterized the resistance of (1) an elastolite, a conductive fabric (MedTex180, SparkFun Electronics, Boulder, CO, USA); (2) a conductive yarn (12 UM Stainless steel fiber with 0.12 mm diameter, SparkFun Electronics, Boulder, CO, USA) and (3) an electric paint (Bare Conductive—Electric Paint, 1.16 g/mL, water-based, SparkFun Electronics, Boulder, CO, USA). The conductive textile was divided in strips of 68 cm of length. The measuring process was the same for all the conductive supports, except the electric paint since it is more complicated to evaluate. We measured different painting strips to determine which one has the best regularity. The best painting strips were used for comparing against the other types of support. Figure 8 presents the resistance of all conductive supports as a function of length.

It shows that the conductive yarn coil has the best conduction, followed closely by the elastolite. This interesting finding could be exploited in a future version of the insole. The main difference between these two technologies is their flexibility: the elastolite cannot be bent in every direction, yet the elastolite and conductive yarn coil are rather close in terms of conductivity, hence can be interchanged depending on the insole’s disposition.

## 3. Experimental Procedure

Due to the higher consumption of the three other types of force sensors, the FSR sensor is a more suitable technology to compute the center of pressure. This section describes the experimental methodology used to validate the proposed measurement system. We present the selected workstation and six series of postures (and movements) that the worker can adopt while accomplishing his or her tasks. We then discuss the representation used for postures (or movements). Finally, the experimental protocol is described.

### 3.1. Workstation and Postures Selected for Data Acquisition

During this phase, we want to represent a posture (or a movement) by the signals collected using our measurement system. Since our aim is to detect inadequate postures during work, this step is important in the learning process where we define the different categories of postures (and movements).

**Figure 9:**
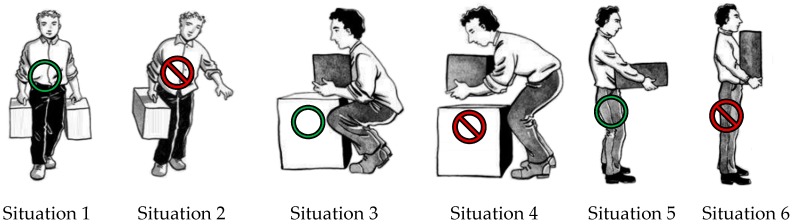
Six situations for evaluating adequate and inadequate posture for handling tasks, this figure is adapted from [53] with permission from Caroline Merola and the publisher.

The workstation consists of the Flexible Manufacturing System (FMS) used by Meziane et al. [52]. It includes a robot with human interaction skills and a Programmable Logic Controller (PLC) connected to the robot, as well as other components such as a conveyor, a distributor and a storage system. The main task of this system is to automatically assemble two metallic pieces (pieces A and B). The assembly task begins by pushing the first piece (piece A) on the conveyor. The size of the pieces is represented in Figure 9. The role of the operator is to fill the distributors with the assembly pieces, and to manage the assembly errors by retrieving the wrongly assembled pieces.

Filling the distributors puts the operator in one of the situations represented in Figure 9. Some of these situations are inadequate, which means that they can lead to WMSDs in the long or medium term. Other positions are adequate, i.e., more secure for WMSD. We referred to Simoneau’s handling manual [53] to select the most frequent adequate and inadequate positions (as shown in Figure 9).

### 3.2. Experimental Protocol

A single participant wore both the helmet (Figure 2) and insole (Figure 3), and simulated twenty trials of each of the situations described in Figure 9. For Situations 1, 2, 5 and 6, to have a good reliability in the information delivered by the COP features while adopting a static position, the duration of the measurement time must vary between 20 and 60 s. In our experiments, we set this duration to 20 s. For Situations 3 and 4, since there are movements, the duration was set to 15 s, i.e., the time needed to perform these movements. Each piece carried by the participant weighed 9 kg, a load considered as moderately heavy. According to Canadian regulations [54], the maximum load that an operator must carry must not exceed 23 kg. In Situations 1 and 2, however, the operator must carry two loads of 9 kg each, the total weight approaching this authorized limit.

**Figure 10:**
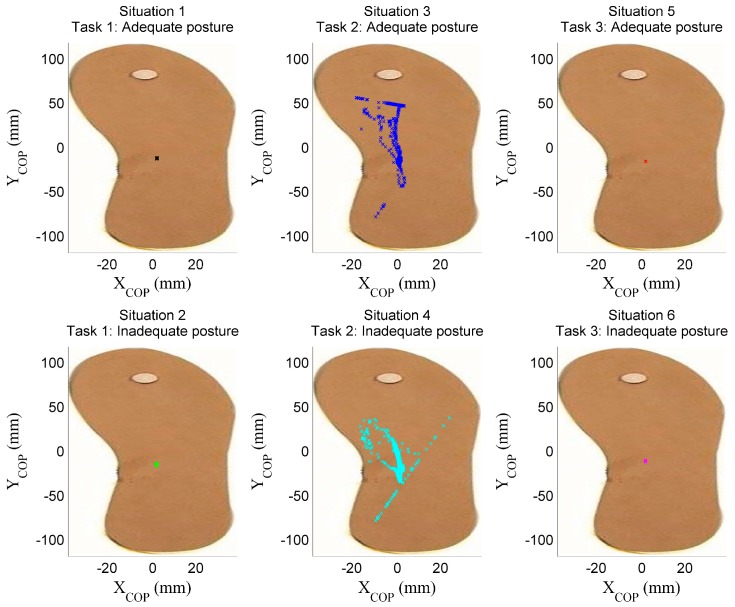
COP’s displacements on the instrumented insole.

**Figure 11:**
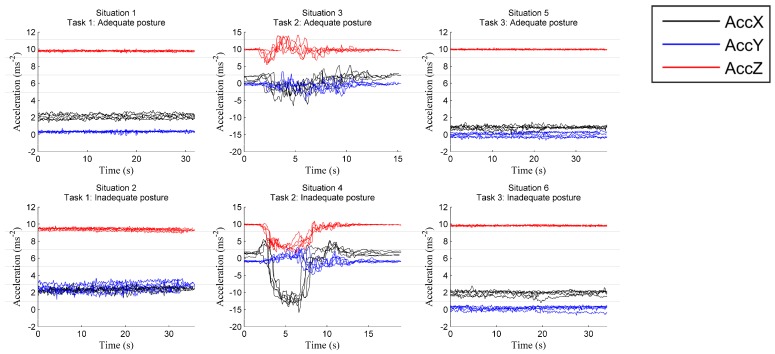
Acceleration signals of the head in three axes (five tests in each case).

The signals related to the situations presented in Figure 9 are acquired by the insole (Figure 10) and the helmet (Figure 11). Figure 10 shows the COP’s displacements on the surface of the insole in the different situations, as computed using Equation (1), and Figure 11 the accelerations of the head captured using the accelerometer of the helmet along the three axes. Visual inspection of the COP dispersion indicates in these figures that Task 2 (Situations 3 and 4) is clearly distinguished from other tasks. This can be explained by the nature of this Task, which keeps the operator moving, whereas a static posture is adopted in the other tasks (Tasks 1 and 3).

Signals from the helmet in Figure 11 show clear differences between the situations of adequate and inadequate posture, these differences more pronounced along the mediolateral (X) and anteroposterior (Y) axes and when the operator is moving (Situations 3 and 4). This can also be seen Table 1, which gives the mean acceleration along axes X and Y for Situations 1, 2, 5 and 6. Moreover, in the case of static posture, the acceleration is nearly constant (the higher value observed for the Z axis is due to gravitation; Z axis is a perpendicular axis to insole surface). In contrast, head accelerations for Task 2 vary sharply due to the operator’s movements during this task.

**Figure 12:**
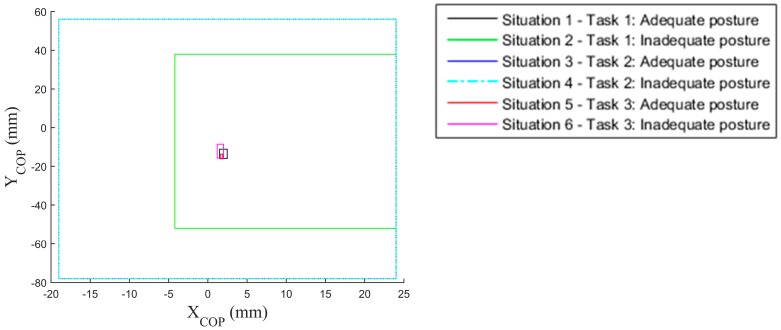
Areas containing the COP displacements measured in different situations.

Figure 10 and Figure 11 present the recordings of a single trial and five trials respectively. To give a more complete view of the data, Figure 12 gives the areas (i.e., bounding boxes) containing the COP displacements of all trials, recorded in each situation. We can note that for adequate postures (zones with black and red borders), the displacement zones are smaller, indicating a certain stability compared to the inadequate static postures (zones with green and pink borders). This observation is not applicable when the operator is in motion (Situations 3 and 4). We can thus use the area of displacement as a criterion to differentiate between specific postures. In this study, we considered the displacements along the mediolateral (Am_X) and anteroposterior (Am_Y) axes, as well as the ellipse of confidence surf_ellip (the area that keeps 90% of the points occupied by the COP).

## 4. Design of the Supervised Model for Posture Classification

**Figure 13:**
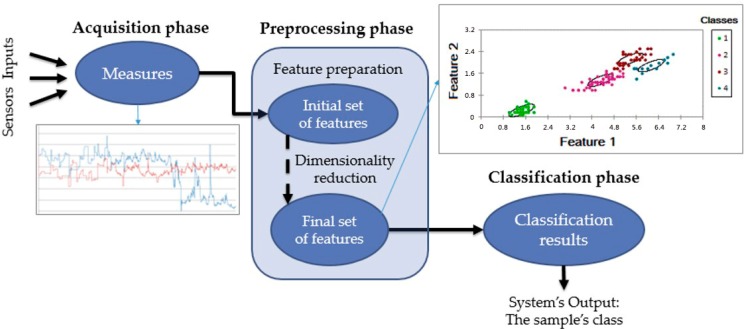
The proposed approach for classifying postures, comprised of three phases: (1) data acquisition; (2) data preprocessing and (3) classification.

The proposed approach for classifying postures is illustrated in Figure 13. It is composed of three distinct phases: (1) Data acquisition, where sample measurements are gathered through a set of sensors; (2) Data preprocessing, which includes features preparation and dimensionality reduction; and (3) Classification, in which the system uses the selected features to evaluate the incoming information and make a final decision as to which class this latter belongs. 

Data acquisition has already been presented in experimental protocol. The preprocessing and classification phases are considered in the following sections.

### 4.1. Feature Preparation

In the processing phase, an initial set of features has to be computed. The goal of this phase is to extract discriminative features from the raw acquired data. Toward this goal, we used two different approaches, a direct approach and a graphic approach, which differ in the nature and the source of extracted features.

#### 4.1.1. The Direct Approach

In this approach, features are determined directly from the helmet and insole recordings. The features extracted from the head acceleration recordings along the three axes AccX, AccY and AccZ are mainly of statistical nature: mean values (i.e., AccXm, AccYm, AccZm), maximum values (i.e., AccXmax, AccYmax, AccZmax), variances (i.e., AccXvar, AccYvar, AccZvar), standard deviations (i.e., AccXstd, AccYstd, AccZstd), root mean squares (i.e., AccXrms, AccYrms, AccZrms), and kurtosis (i.e., AccXkurt, AccYkurt, AccZkurt).

Features extracted from COP recordings are based on two different representations: the statokinesigram (COP trajectory in the horizontal plane) and the stabilogram (variations of the time series showing the anteroposterior Y_COP_ and the mediolateral X_COP_). Various features can be obtained from these representations to analyze the control postural while adopting a certain posture. Among these are global variables [36], which characterize the magnitude of *X*_COP_*,* Y_COP_ and their resultant in both time and frequency domains [55,56]. These variables are widely used in the literature for various applications, including postural control in aging (such as Parkinson’s and ataxia subjects) [55,57]. Features may also be defined from structural variables, which describe the dynamic changes of postural sway by decomposing the COP sway patterns into subunits and correlating them with the motor control process [55,56]. These variables are particularly useful to cover the non-stationary character of the COP displacement. Examples of structural variables are those proposed by Collins and De Luca [58], which are based on the Stabilogram Diffusion Analysis method. This method models the trajectory of the COP using stochastic processes such as the fractional Brownian motions. The Fractal Analysis developed by Blaszczyk [59] also provides other structural variables related to postural perturbations. In the context of this work, we limited our selection of features to the set of global variables shown in Table 2. These features were grouped into three categories: spatiotemporal data extracted from the statokinesigram, spatiotemporal data extracted from the stabilograms and frequency data.

#### 4.1.2. The Graphic Approach

**Figure 14:**
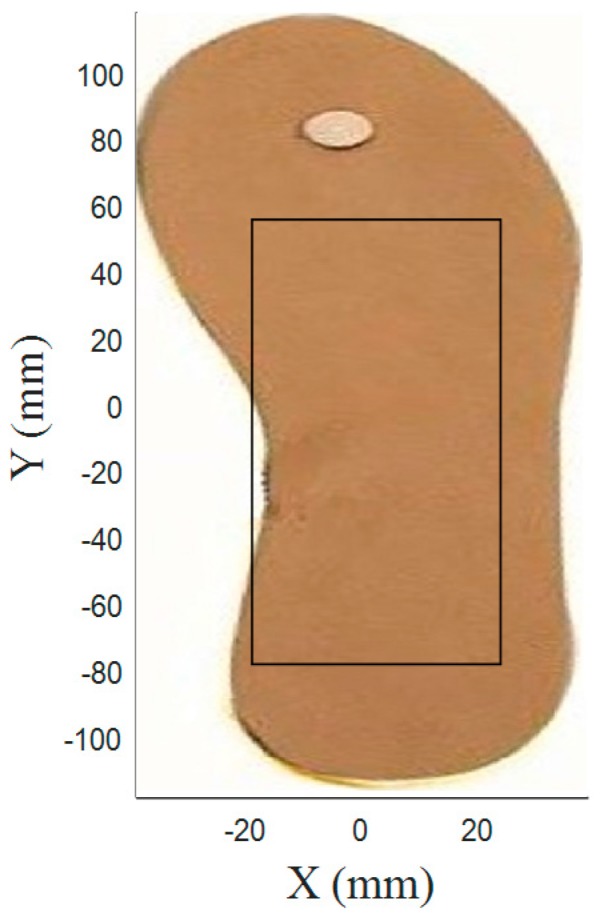
Representation of the area of the insole occupied by the COP.

**Figure 15:**
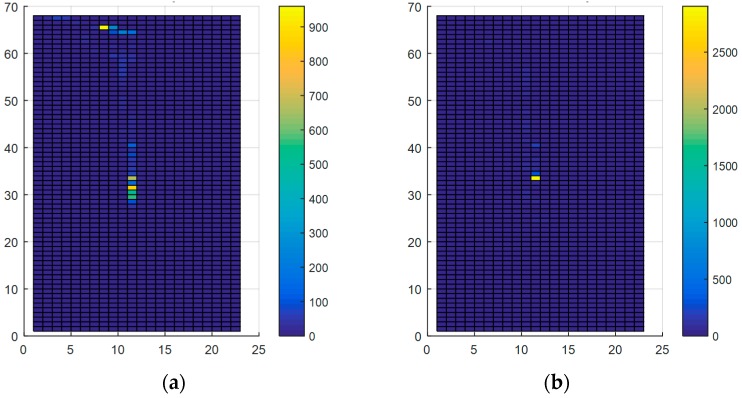
The matrix of pixels in two different situations with a resolution of 1564: (**a**) Adequate posture in Task 2; (**b**) Inadequate posture in Task 2.

To acquire additional characteristics for differentiating between postural Situations, we also proposed a graphic approach which represents COP displacements from the insole as an image (matrix of pixels). To fix the surface of the insole to be discretized, we first computed the minimum/maximum values of X_COP_ and Y_COP_ across all measurements. The resulting area, shown in Figure 14, has a total size of 43 × 134 mm^2^ and is discretized using different resolutions (i.e., number of pixels in the image). Table 3 gives the correspondence between the resolution and the corresponding area of a single matrix element. The features obtained by this method are the elements of the matrix representing the area of the insole that has been occupied by the center of pressure at least once. Figure 15 shows an example of matrices obtained using this method.

### 4.2. Dimensionality Reduction

Dimensionality reduction is an important step of classification systems to select an optimal set of features and limit the complexity of the model [60]. There are two main types of methods for reducing the dimensionality, based on feature transformation and feature selection. Feature transformation methods like Principal Component Analysis (PCA) and the Linear Discriminant Analysis (LDA) project the initial features into a new space with lower dimensionality. While feature transformation generates a new set of features by combining existing ones, feature selection methods simply reduce the initial set of features to those most useful for classification [61,62,63,64]. The advantage of this method is that it allows keeping the semantics of the initial features thereby facilitating the analysis of results. In our study, we considered two popular features selection models: the Filter Model and the Wrapper Model.

#### 4.2.1. The Filter Model

We performed a selection of features, with five commonly-used filter techniques presented in Table 4. These techniques are based on statistical tests, which evaluate the importance of a feature for discriminating between classes. The scores of each feature (see Table A1) are first measured using each of these statistical tests, and normalized to a value between 0 and 1. Since the importance of features may vary from one test to another, we combine these scores into a single value by taking their average for each feature. Using this aggregate score, features are then ranked and the highest ranked ones are kept for classification. Note that this feature selection step is independent of the model used for classification. Table A2 shows the selected features according to each method, and using the combination of all statistical tests. The disadvantage of this approach is that it does not consider interactions between features and, thus, there may be information redundancy in the final subset of features.

#### 4.2.2. The Wrapper Model

Unlike the filter one, the wrapper model selects features according to the accuracy of a classifier. As its name suggests, the classifier’s training algorithm is “wrapped” inside the feature selection process, making it this approach suitable for any classifier. However, since the classifier has to be trained and evaluated several times, it is costlier in terms of time and calculation. The wrapper model chosen in this work is the Sequential Forward Selection (SFS) [63]. This model starts with an initial set of features regrouping the preselected features of the filter model and a final set of features initially empty. The operation consists of filling the final set of features with the sorted features of the initial set. The sorting process is performed as follows: it iteratively adds to the final set, the feature that gives the best classification result when combined to the actual final set. The model stops once the final set is filled with all the initial features. In our experiments, we applied the SFS feature selection using the wrapper model on the intermediate subsets kept at the previous step (the first twenty features kept by the dir_filter_1 and dir_filter_6 methods in Table A2).

#### 4.2.3. The Hybrid Feature Selection

**Figure 16:**
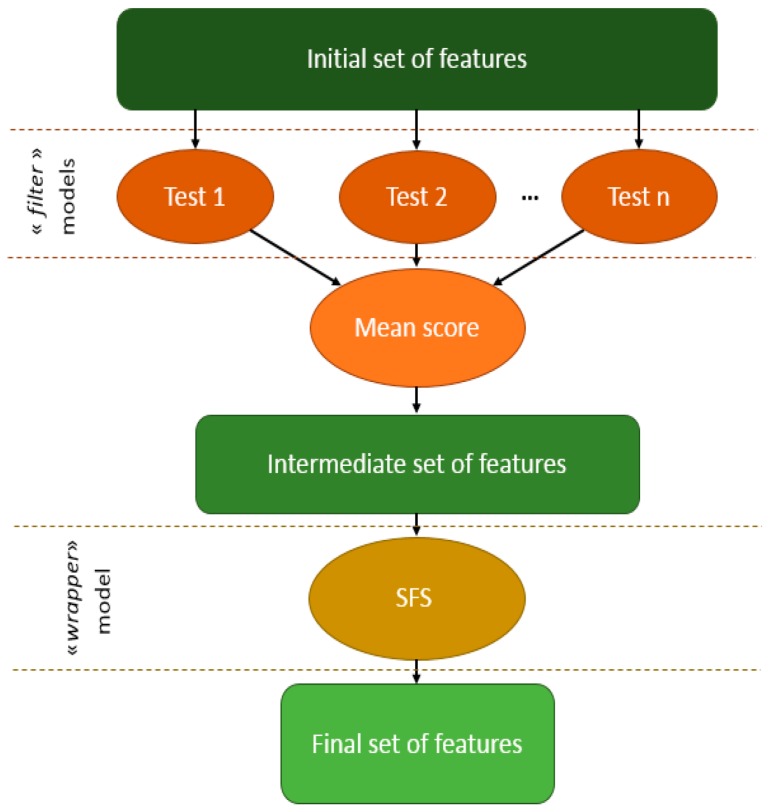
The hybrid model for feature selection.

This method, illustrated in Figure 16, corresponds to the successive application of the filter model followed by the wrapper model. These models work in a complementary way, the former being faster but less reliable (mainly because of its independence from the classifier) than the latter. Here, we use the filter model to do a preselection of important features, and then apply the wrapper model on this intermediate set for selecting the final best features. This hybrid feature selection is applied on all the features obtained with the direct approach and the graphic approach during the feature preparation process. Using the hybrid feature selection method, the 60 direct approach features (Table A1) have been reduced to eight (Table 5) and the 990 graphic approach features to three.

### 4.3. Classification Phase

After feature selection, we proceeded with the classification process. Given the recent successes of Artificial Neural Networks (ANN), in this work, we chose a multi-layer perceptron as classifier. Our network is organized as follows:

- An input layer of i neurons, where i is the number of inputs (i.e., final set features);
- A hidden layer of j = 12 neurons whose activation function is the hyperbolic tangent;
- An output layer of k = 6 neurons, where k is the number of classes (posture situations presented previously). A softmax function is used to convert the output of these neurons into class probabilities [67].

As in most classification networks [68,69], cross-entropy is used as loss function:

(2) $$E=\frac{1}{l}\overset{l}{\underset{j=1}{\sum }}\overset{k}{\underset{n=1}{\sum }}T_{\mathrm{ij}}\ln\left(S_{\mathrm{ij}}\right)$$

where k represents the number of classes, l represents the total number of sample groups and T_ij_ is the desired response of the output neuron i of the sample group j. The specificity of this function is that it strongly penalizes the wrong outputs (whose values are far from the desired value T_ij_ and thus closer to (1−T_ij_)) and emphasize those that are correct [69]. As a learning algorithm, we adopted the conjugate gradient method. This method is appropriate for classification problems when the volume of data is not very large [68].

## 5. Classification Results and Discussion

Bestaven et al. [70] show that the total center of pressure (TCOP) displacements, initially located between the two feet, seems to be a good tool to identify some strategies but during the sit-to-walk especially in the elderly. Several studies cited in [42,43] have shown that COP does not differ between dominant and non-dominant limbs. Thereby, in our study, we therefore believe that no effect can be observed on the results of measurement by both feet. The goal of this study is to use a minimal configuration and architecture allowing the classification of six postures in order to develop an inexpensive assistance device which can be used at work in real environment. This system is mainly designed for a long-term monitoring of worker. Then, using one insole is an efficient solution giving an adequate classification of postures presented below:

### 5.1. Application of the Filter Technique on the Direct Approach Features

In Table A2, we notice that most of the tests kept more or less the same features in their top ten lists, which allowed us to keep the overall score naturally. The only exception is the Pearson test (dir_filter_2 method), which gave one of the poorest classification performances. We tested the different sets of features on a neural network, using a 10-fold cross validation technique. For more reliable results, this cross-validation process was repeated 10 times, and the average classification rate over these 10 runs used as final performance measure.

**Figure 17:**
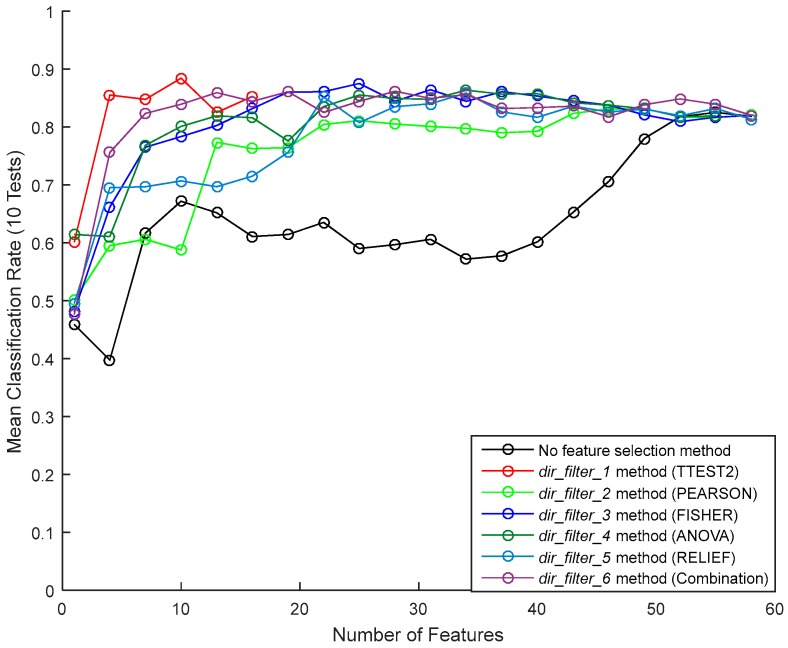
Averages of the recognition rates obtained by the filter selection method.

Figure 17 shows the mean classification rate of the feature selection techniques, for different numbers of selected features. We notice a poor performance when no feature selection is used. For most feature selection methods, classification rates reach a peak performance (around 80%) between 20 and 30 features, this performance remaining stable for larger numbers of feature. To have an efficient classifier, we limit the number of features to 20. Using this limit, Ttest2 (dir_filter_1 method) gave the best classification result, followed by the combination of all the scores (dir_filter_6 method). Therefore, we kept the first 20 features of these two methods as intermediate subsets of features for the wrapper technique.

### 5.2. Application of the Hybrid Feature Selection on the Direct Approach Features

**Figure 18:**
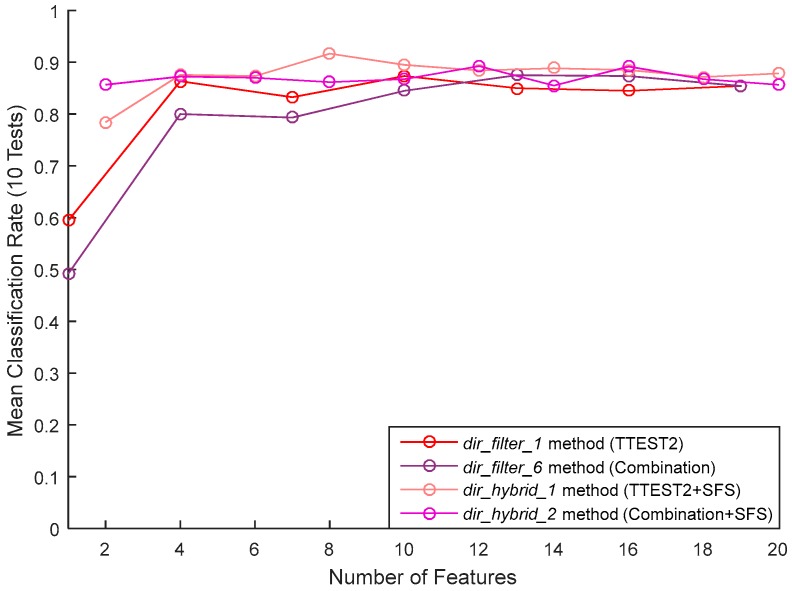
Comparison between the performances of the filter and the hybrid selection methods.

Table A3 gives the importance-sorted list of features obtained with the dir_hybride_1 and dir_hybride_2 methods (i.e., dir_filter_1 and dir_filter_6 technique, respectively, followed by the SFS wrapper). The classification results of these two methods are shown in Figure 18. To compare the hybrid feature selection method with using the filter method alone, and highlight the contribution of the wrapper model, the figure also gives the network’s performance corresponding to the features kept by dir_filter_1 and dir_filter_6. For both these filter models, we notice an increase in performance when going from the filter alone to the hybrid model. Comparing the two hybrid methods, no significant difference in performance can be seen. Overall, a best performance of 90% was obtained by the dir_hybride_1 method using only eight features.

### 5.3. Application of the Hybrid Feature Selection on the Graphic Approach Features

**Figure 19:**
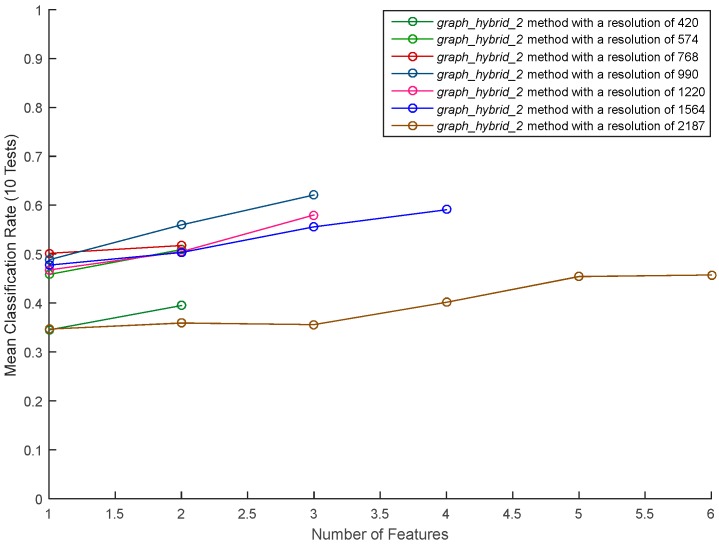
Performance of the neural network with the graphical method according to different resolutions.

We applied dir_hybride_1 and dir_hybride_2 (described in Table A3) to the graphic approach features, the resulting methods called graph_hybride_1 and graph_hybride_2, respectively. Since both these methods selected the same features (with similar feature rankings), we only show in Figure 19. the classification results of graph_hybride_2. These results indicate that using the graphic approach features alone leads to poor performances, with a best classification rate near 60% (matrix resolution of 990). In the next section, we improve our results by combining these features with those of the direct approach.

### 5.4. Combination of Direct and Graph Approach Features

**Figure 20:**
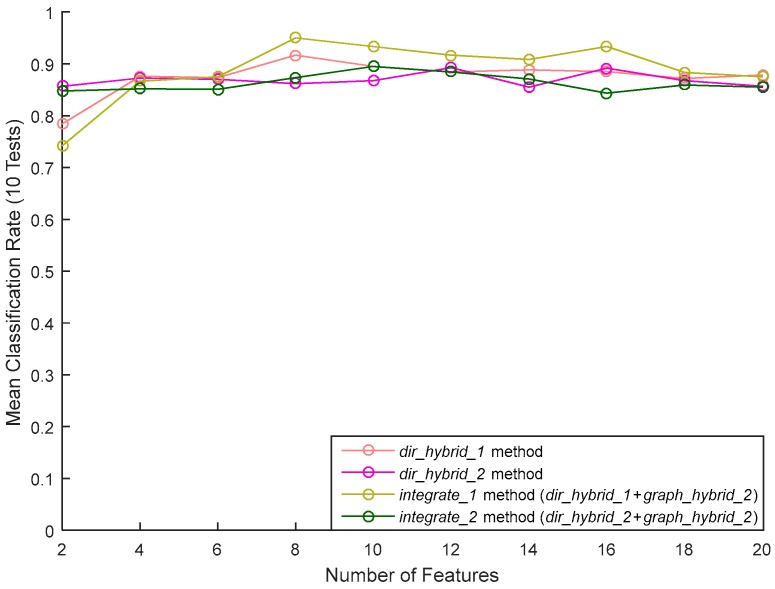
The integration of the direct and graphical methods.

We added the three best features of the graphic approach, obtained by applying the graph_hybride_1 method, to the features of the direct approach selected via the dir_hybride_1 and dir_hybride_2 methods (shown previously in Figure 18). We name integrate_1 and integrate_2, respectively, these two combinations of features. The classification results of these methods are shown in Figure 20. It can be seen that combining features improves results, the classification rate now reaching 95% when using the eight best features (presented by order of importance in Table 5) of dir_hybride_1 with the three best features of graph_hybride_1. We note that this result is achieved with only 11 features from the initial set of 1050 features.

## 6. Conclusions and Future Works

This study aimed to design an automated assessment tool for posture analysis. Using specific features, we demonstrated that artificial neural networks (ANN) can effectively be used to detect inadequate postures of individuals in a work environment. A direct approach was used to collect features from different postures and movements. We also introduced an innovative graphic approach, which represents movements of the center of pressure, captured by the insole, as an image. A hybrid model was used to select the most discriminative features generated by these two approaches. An important contribution of this study was to combine features from both approaches for the effective identification and prevention of inadequate postures. The proposed methodology allowed us to improve the recognition rate from 90% to 95%, an accuracy superior to that of existing models.

A limitation of this study is the small sample size, which was obtained using a single participant. Although our results suggest that the proposed method can be used successfully to assess work-related MSDs, testing on a larger dataset with multiple participants would help to further validate these results. Future research can also apply the Graphic approach to extract features from the helmet recordings. Toward this goal, measurements of the IMU can be exploited to determine the trajectories of the head in the planes formed by its three axes of motion, namely the plane (X, Y), the plane (Y, Z) and the plane (X, Z). Subsequently, as with COP displacements, we can use three matrices representing these trajectories. We also plan to integrate other inertial units located in areas of the body, such as the back and the upper/lower limbs. Adding these new sensors in other locations may improve the accuracy of the system. In addition, these sensors (specifically those in the upper limbs) may detect repetitive movements, which are one of the major risk factors aside from awkward postures and movements. Finally, we can extend our detection system to significant muscle efforts, for instance, detected via an electromyogram (EMG).

## Figures and Tables

**Table 1:** Approximation of the mean values of the head accelerations according the mediolateral (X) and the anteroposterior (Y).

Mean Value (ms^−2^)	Situation 1	Situation 2	Situation 5	Situation 6
**AccX**	≈2	≈2	≈1	≈2
**AccY**	≈0	≈2	≈0	≈0

**Table 2:** List of the features extracted from the COP recordings (insole only).

Type	Description	Name
**Spatiotemporal features extracted from the statokinesigram**	The total length of the statokinesigram	lg_tot
	The mean and standard deviation of the statokinesigram’s segments lengths	m_lg_seg std_lg_seg
	The distance between the first and last point of the statokinesigram	dist_prdr
	The amplitude of the displacement in the mediolateral (X_COP_) and anteroposterior (Y_COP_) axes	Am_X Am_Y
	The surface of the ellipse covering 90% of the displacements of the COP	surf_ellip
	The length/surface ratio, which informs us on the energy spent by the subject during the postural control	lfs
**Spatiotemporal features extracted from the stabilograms**	The mean and maximum values, the variances, the standard deviations, the root-mean-squares and the kurtosis of the displacements in the mediolateral (X_COP_) and anteroposterior (Y_COP_) axes.	Xm, Ym, Xmax, Ymax Xstd, Ystd, Xvar, Yvar Xrms, Yrms, Xkurt, Ykurt
	Statistical data related to the velocity (global, mediolateral and anteroposterior) such as mean and maximum values, the variances, the standard deviations, the root-mean-squares and the kurtosis	Vm, VXm, Vym Vmax, VXmax, Vymax Vstd, VXstd, Vystd Vvar, Vxvar, Vyvar Vrms, VXrms, Vyrms Vkurt, VXkurt, VYkurt
**Frequential features**	Mean and median frequencies of the mediolateral (X_COP_) and anteroposterior (Y_COP_) displacements	mnfreqX, mnfreqY mdfreqX, mdfreqY

**Table 3:** List of the tested resolutions and their correspondent superficies.

Resolution (Number of Matrix Elements)	Area Represented by a Single Matrix (mm^2^)
420	3.50 × 3.82
574	3.07 × 3.26
768	2.68 × 2.79
990	2.38 × 2.43
1220	2.15 × 2.19
1564	1.86 × 1.97
2187	1.59 × 1.65

**Table 4:** The filter techniques and their characteristics.

Name	Description	Equation
**Fisher criterion**	It represents one of the most common *«filter»* models used to select features [61,65]. This criterion emphasizes the importance of each feature by calculating the ratio of separation between two classes with respect to their dispersions. With this criterion, the features with the highest results allow a better discrimination between the classes	$F\left(i\right)=\frac{{\left(\mu_{2}\left(i\right)-\mu_{1}\left(i\right)\right)}^{2}}{\left({\left(\sigma_{1}\left(i\right)\right)}^{2}+{\left(\sigma_{2}\left(i\right)\right)}^{2}\right)}$ $\mu_{j}\left(i\right)$ and $\sigma_{j}\left(i\right)$ represent respectively the mean and the standard deviation of the ith feature of the class j
**Two-sample *t*-test **	This *«filter»* approach is also widely used to estimate the importance of each feature in discriminating between the different classes [61]. The two-sample *t*-test, is a univariate statistical test that analyses whether we could consider two independent samples as coming from classes with unequal means by analyzing the values of the given feature	$t\left(i\right)=\frac{\mu_{2}\left(i\right)-\mu_{1}\left(i\right)}{\sqrt{{\left(\frac{\sigma_{1}\left(i\right)}{n}\right)}^{2}+{\left(\frac{\sigma_{2}\left(i\right)}{m}\right)}^{2}}}$ n and m represent respectively the number of samples of the classes 1 et 2. They are equal in our case
**Pearson Correlation Coefficient **	The correlation coefficient is also used to determine the discrimination power of each features between the different classes [64,66]. The result of the equation gives us an idea about the degree of similarity between two classes. It varies between −1 and 1. The closer it is to 0, the more insignificant the relationship between classes is, and the closer it gets to 1 or −1, the more significant it is	$R\left(i\right)=\left(\frac{1}{n}\right)\frac{\sum_{j}^{n}\left(x_{1}^{j}-\mu_{1}\left(i\right)\right)\left(x_{2}^{j}-\mu_{2}\left(i\right)\right)}{\sigma_{1}\left(i\right)\times \sigma_{2}\left(i\right)}$ $x_{1}^{j}$ and $x_{2}^{j}$ represent the jth samples of the classes 1 and 2 respectively
**ANOVA**	The analysis of variance aims to test the significant differences between the means, it represents an extension of the Ttest2 for multi-class problems. We used this technique to test whether or not a feature allows a good discrimination between the different classes of the problem	-
**Relief**	The Relief technique makes it possible to measure the relevance of the features by accumulating the difference of the distances between randomly selected learning variables and their closest neighbors of the same class, and subtracting the distances with the variables of the other classes [62,63]. We used the generalized version of this method, named ReliefF, used for multi-class problems	-

**Table 5:** List of the best «Direct» features obtained with the method dir_hybride_1 (dir_filter_1 (Test2) and wrapper).

Id	Name	Description	Tool
55	AccZm	Mean acceleration of the head along the axis Z	Helmet
10	Yvar	Y_COP_ variance	Insole
51	AccYstd	Standard deviation of the acceleration along the axis Y	Helmet
47	AccXrms	The root mean square of the acceleration along the axis X	Helmet
3	Xstd	Standard deviation of *X_COP_*	Insole
49	AccYm	Mean acceleration of the head along the axis Y	Helmet
44	AccXmax	Maximal head acceleration along the axis X	Helmet
52	AccYvar	Variance of the head acceleration along the axis Y	Helmet

## Appendix A

**Table A1:** Scores obtained by the filter feature selection methods.

Id	Name	Anova (*p*-Value)	Ttest2 (*p*-Value)	Pearson (Score)	Fisher (Score)	Relief (Score)	Final (Score)
1	Xm	8.43 × 10^−32^	1.06 × 10^-1^	2.64 × 10^−2^	2.63	1.84 × 10^−1^	0.443
2	Xmax	3.55 × 10^−24^	1.10 × 10^−1^	−6.51 × 10^−2^	1.90	2.97 × 10^−1^	0.338
3	Xstd	3.88 × 10^−54^	3.53 × 10^−2^	−5.27 × 10^−2^	7.01	2.79 × 10^−1^	0.324
4	Xvar	6.28 × 10^−40^	1.30 × 10^−1^	−4.98 × 10^−2^	3.97	2.04 × 10^−1^	0.394
5	Xrms	8.03 × 10^−29^	9.36 × 10^−2^	−5.45 × 10^−2^	2.06	1.61 × 10^−1^	0.399
6	Xkurt	5.05 × 10^−1^	2.28 × 10^−1^	−4.26 × 10^−2^	6.28 × 10^−1^	6.22 × 10^−3^	0.625
7	Ym	1.16 × 10^−57^	2.47 × 10^−2^	−6.02 × 10^−2^	11.20	2.38 × 10^−1^	0.331
8	Ymax	1.03 × 10^−75^	1.46 × 10^−1^	2.10 × 10^−2^	2.83 × 10^2^	4.56 × 10^−1^	0.135
9	Ystd	2.21 × 10^−93^	2.64 × 10^−3^	5.44 × 10^−2^	40.5	3.34 × 10^−1^	0.330
10	Yvar	1.08 × 10^−66^	2.18 × 10^−2^	9.64 × 10^−3^	11.10	2.23 × 10^−1^	0.379
11	Yrms	1.09 × 10^−69^	1.82 × 10^−2^	7.27 × 10^−32^	10.90	2.32 × 10^−1^	0.413
12	Ykurt	4.06 × 10^−1^	2.89 × 10^−1^	2.11 × 10^−1^	3.79 × 10^−1^	4.13x10^−3^	0.784
13	Vm	1.45 × 10^−48^	1.22 × 10^−1^	5.01 × 10^−4^	5.52	2.41 × 10^−1^	0.405
14	Vmax	1.04 × 10^−19^	1.69 × 10^−1^	4.47 × 10^−2^	2.91	9.14 × 10^−2^	0.517
15	Vstd	4.16 × 10^−34^	1.79 × 10^−1^	−8.17 × 10^−3^	3.08	1.52 × 10^−1^	0.460
16	Vvar	9.64 × 10^−35^	1.50 × 10^−1^	5.97 × 10^−3^	3.19	1.55 × 10^−1^	0.457
17	Vrms	4.33x10^−11^	1.87 × 10^−1^	−7.68 × 10^−3^	8.34 × 10^−1^	4.84 × 10^−2^	0.511
18	Vkurt	4.50x10^−5^	2.10 × 10^−1^	4.93 × 10^−2^	3.72 × 10^−1^	4.52 × 10^−2^	0.556
19	VXm	9.86 × 10^−1^	5.00 × 10^−1^	3.55 × 10^−2^	3.59 × 10^−2^	4.98 × 10^−3^	0.867
20	VXmax	1.60 × 10^−17^	2.16 × 10^−1^	3.50 × 10^−2^	2.41	6.11 × 10^−2^	0.541
21	VXstd	2.74 × 10^−31^	2.22 × 10^−1^	−2.70 × 10^−2^	2.89	1.07 × 10^−1^	0.484
22	VXvar	2.78 × 10^−31^	2.22 × 10^−1^	−2.70 × 10^−2^	2.89	1.07 × 10^−1^	0.484
23	VXrms	2.51 × 10^−9^	2.46 × 10^−1^	−1.08 × 10^−2^	8.32 × 10^−1^	2.26 × 10^−2^	0.541
24	VXkurt	1.72 × 10^−9^	2.28 × 10^−1^	−1.52 × 10^−2^	6.61 × 10^−1^	6.78 × 10^−2^	0.512
25	Vym	9.76 × 10^−1^	5.70 × 10^−1^	6.22 × 10^−3^	2.76 × 10^−2^	7.69 × 10^−3^	0.871
26	VYmax	3.30 × 10^−15^	1.94 × 10^−1^	2.56 × 10^−2^	1.14	5.99 × 10^−2^	0.529
27	VYstd	2.48 × 10^−31^	1.59 × 10^−1^	1.28 × 10^−2^	2.50	1.45 × 10^−1^	0.470
28	VYvar	2.52 × 10^−31^	1.59 × 10^−1^	1.29 × 10^−2^	2.50	1.45 × 10^−1^	0.470
29	VYrms	2.13 × 10^−9^	1.58 × 10^−1^	1.52 × 10^−2^	7.00 × 10^−1^	4.49 × 10^−2^	0.517
30	VYkurt	1.12 × 10^−7^	1.81 × 10^−1^	4.79 × 10^−2^	5.48 × 10^−1^	6.13 × 10^−2^	0.538
31	long_tot	1.54 × 10^−45^	9.67 × 10^−2^	4.98 × 10^−3^	4.42	1.92 × 10^−1^	0.421
32	m_long_seg	6.90 × 10^−50^	1.03 × 10^−1^	6.50 × 10^−3^	5.51	2.33 × 10^−1^	0.405
33	std_long_seg	2.78 × 10^−44^	1.26 × 10^−1^	2.96 × 10^−2^	4.19	2.10 × 10^−1^	0.439
34	dist_prdr	2.39 × 10^−2^	1.55 × 10^−1^	4.08 × 10^−2^	2.62 × 10^−1^	7.15 × 10^−3^	0.553
35	plage_X	1.79 × 10^−53^	7.59 × 10^−2^	−4.99 × 10^−2^	7.52	3.88 × 10^−1^	0.291
36	plage_Y	2.14 × 10^−59^	9.94 × 10^−2^	2.05 × 10^−1^	12.10	3.28 × 10^−1^	0.480
37	surf_ellip	5.65 × 10^−49^	4.67 × 10^−2^	−3.49 × 10^−2^	5.40	2.36 × 10^−1^	0.359
38	Lfs	8.33 × 10^−9^	2.40 × 10^−2^	6.83 × 10^−2^	6.18 × 10^−1^	3.39 × 10^−2^	0.507
39	mnfreqX	4.08 × 10^−29^	1.47 × 10^−1^	−2.06 × 10^−2^	2.06	1.40 × 10^−1^	0.448
40	mnfreqY	9.05 × 10^−31^	7.43 × 10^−2^	1.45 × 10^−1^	2.88	1.37 × 10^−1^	0.525
41	mdfreqX	7.60 × 10^−29^	7.92 × 10^−2^	9.55 × 10^−3^	2.19	1.27 × 10^−1^	0.448
42	mdfreqY	4.83 × 10^−31^	3.74 × 10^−2^	1.31 × 10^−1^	2.83	1.19 × 10^−1^	0.511
43	AccXm	2.19 × 10^−40^	7.51 × 10^−2^	1.62 × 10^−1^	6.33	2.05 × 10^−1^	0.503
44	AccXmax	1.94 × 10^−34^	7.24 × 10^−2^	8.73 × 10^−2^	4.36	1.94 × 10^−1^	0.462
45	AccXstd	2.21 × 10^−95^	6.56 × 10^−2^	−1.89 × 10^−2^	66.30	3.24 × 10^−1^	0.293
46	AccXvar	2.77 × 10^−60^	9.76 × 10^−2^	6.33 × 10^−2^	2.16 × 10^1^	2.33 × 10^−1^	0.427
47	AccXrms	1.12 × 10^−87^	5.78 × 10^−2^	−4.74 × 10^−2^	2.01 × 10^1^	2.55 × 10^−1^	0.336
48	AccXkurt	7.37 × 10^−2^	3.33 × 10^−1^	1.73 × 10^−2^	1.55 × 10^−1^	8.31 × 10^−3^	0.611
49	AccYm	1.74 × 10^−42^	8.42 × 10^−4^	−2.31 × 10^−2^	6.46	2.02 × 10^−1^	0.364
50	AccYmax	5.61 × 10^−31^	9.43 × 10^−3^	−9.55 × 10^−2^	4.48	1.47 × 10^−1^	0.349
51	AccYstd	3.04 × 10^−72^	3.26 × 10^−4^	−3.65 × 10^−2^	17.80	3.34 × 10^−1^	0.289
52	AccYvar	4.14 × 10^−48^	3.44 × 10^−2^	−7.64 × 10^−5^	9.98	2.23 × 10^−1^	0.379
53	AccYrms	6.35 × 10^−54^	1.46 × 10^−3^	−3.38 × 10^−2^	6.62	2.66 × 10^−1^	0.330
54	AccYkurt	5.93 × 10^−1^	3.47 × 10^−1^	−1.15 × 10^−1^	1.86 × 10^−1^	6.84 × 10^−3^	0.641
55	AccZm	1.15 × 10^−66^	3.23 × 10^−2^	5.87 × 10^−3^	1.55 × 10^1^	3.14 × 10^−1^	0.337
56	AccZmax	7.20 × 10^−32^	1.06 × 10^−1^	8.16 × 10^−2^	8.62	1.86 × 10^−1^	0.471
57	AccZstd	1.45 × 10^−72^	2.44 × 10^−3^	−8.06 × 10^−2^	19.90	3.18 × 10^−1^	0.268
58	AccZvar	1.29 × 10^−70^	1.06 × 10^−3^	5.93 × 10^−2^	18.00	2.61 × 10^−1^	0.381
59	AccZrms	7.42 × 10^−53^	2.66 × 10^−3^	−6.87 × 10^−2^	6.41	2.19 × 10^−1^	0.330
60	AccZkurt	7.72 × 10^−8^	3.32 × 10^−1^	1.18 × 10^−1^	6.57 × 10^−1^	6.09 × 10^−2^	0.634

**Table A2:** Features kept by the filter feature selection methods.

Method		Features Kept (from the Most Relevant to the Least))
Name	Test	
dir_filter_1	Ttest 2 (*p*-value ≥ 0.05)	51-49-58-53-57-9-59-50-11-10-38-7-55-52-3-42-37
dir_filter_2	Pearson	52-13-31-55-16-25-32-17-15-41-10-23-27-28-29-24-48-45-39-8-49-26-1-21-22-33-53-37-20-19-51-34-6-14-47-30-18-4-35-3-9-5-58-7-46-2-38-59-11-57-56-44-50-54-60-42-40-43-36-12
dir_filter_3	Fisher	8-45-9-46-47-57-58-51-55-36-7-10-11-52-56-35-3-53-49-59-43-13-32-37-50-31-44-33-4-16-15-14-21-22-40-42-1-27-28-20-41-5-39-2-26-17-23-29-24-60-6-38-30-12-18-34-54-48-19-25
dir_filter_4	Anova (*p*-value ≥ 0.05)	45-9-47-8-57-51-58-11-10-55-46-36-7-3-53-35-59-32-37-13-52-31-33-49-43-4-16-44-15-56-1-27-28-21-22-42-50-40-39-41-5-2-14-20-26-17-24-29-23-38-60-30-18-34-48
dir_filter_5	Relief	8-35-51-9-36-45-57-55-2-3-53-58-47-13-7-37-32-46-11-10-52-59-33-43-4-49-44-31-56-1-5-16-15-50-27-28-39-40-41-42-21-22-14-24-30-20-60-26-17-18-29-38-23-48-25-34-54-6-19-12
dir_filter_6	Combination (combination of all tests)	8-57-51-35-45-3-59-53-9-7-47-55-2-50-37-49-52-10-58-4-5-13-32-11-31-46-33-1-39-41-16-15-44-27-28-56-36-21-22-43-38-42-17-24-14-29-40-26-30-20-23-34-18-48-6-60-54-12-19-25

**Table A3:** Features kept with the hybrid feature selection methods.

Name	Description	Features Kept (from the Most Relevant to the Least)
dir_hybride_1	dir_filter_1 (Ttest2) + wrapper (SFS)	55-10-51-47-3-49-44-52-9-45-59-42-53-37-7-57-58-50-38-11
dir_hybride_2	dir_filter_6 (Combination) + wrapper (SFS)	10-49-58-7-9-37-51-45-52-3-31-53-59-55-13-32-57-35-47-8
